# Silver encapsulated copper salen complex: efficient catalyst for electrocarboxylation of cinnamyl chloride with CO_2_†

**DOI:** 10.1039/c9ra05253d

**Published:** 2019-10-15

**Authors:** La-Xia Wu, Ying-Guo Zhao, Ye-Bin Guan, Hui Wang, Yang-Chun Lan, Huan Wang, Jia-Xing Lu

**Affiliations:** AnHui Province Key Laboratory of Functional Coordination Compounds, School of Chemistry and Chemical Engineering, Anqing Normal University Anqing 246011 China; School of Chemistry and Molecular Engineering, East China Normal University Shanghai 200062 China hwang@chem.ecnu.edu.cn jxlu@chem.ecnu.edu.cn +86-21-52134935 +86-21-62233491; Department of Electrical and Electronic Engineering, Southern University of Science and Technology ShenZhen 518055 China

## Abstract

An active catalyst, [Cu]@Ag composite, was synthesized for the first time and used as a cathode for electrocarboxylation of cinnamyl chloride with CO_2_. β,γ-Unsaturated carboxylic acids were obtained with excellent yield and moderate selectivity. Moreover, reasonable yields and selectivities of carboxylic acids were also achieved with several allylic halides and aryl halides.

## Abstract 2

An active catalyst, [Cu]@Ag composite, was synthesized for the first time and used as a cathode for electrocarboxylation of cinnamyl chloride with CO_2_.

## Graphical abstract

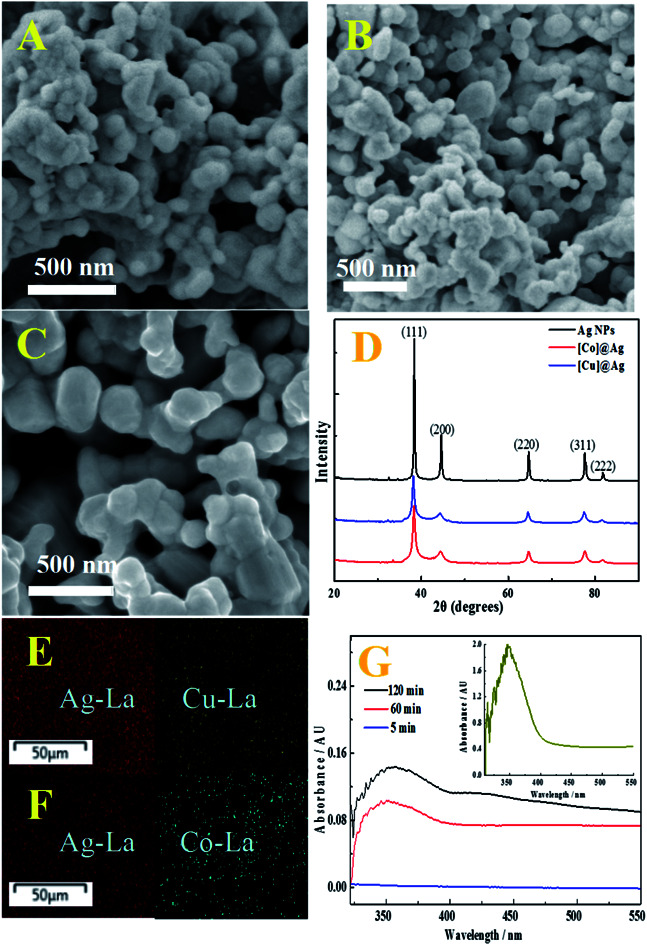

The utilization of CO_2_ has great potential in the field of environmental protection and energy development. Therefore, a number of strategies for CO_2_ conversion are being developed, despite its thermodynamic stability and low reactivity,^1–7^ especially in the transformation of CO_2_ to carboxylic acids. As far as we know, the allylation reaction of CO_2_ would be an efficient approach for the synthesis of β,γ-unsaturated carboxylic acids with unique biological activities. In earlier research, β,γ-unsaturated carboxylic acids were synthesized by organometallic nucleophiles such as allylic lithium and Grignard reagents. However, this process generates a large amount of waste reagents.^8,9^ Transition metal-catalyzed allylation of CO_2_ with allyl acetates^10^/allylic alcohols^11–13^ (Table S1,† entries 1–4), also yields β,γ-unsaturated carboxylic acids, which is mediated by super-stoichiometric amounts of reducing agents (Et_2_Zn,^12^ Mn (ref. 10, 11 and 13)). Recently, Ma and co-workers have successfully developed indium-mediated allylation of CO_2_ starting directly from allylic halides at 60 °C with 2.0 MPa pressure of CO_2_ (Table S1,† entry 5). However, the reaction conditions were not very mild.^14^ Subsequently, they reported direct carboxylation of allylic bromides with CO_2_ with excess amounts of Zn reductant^15^ (Table S1,† entry 6). More recently, the Mei group reported Pd-catalyzed regioselective electrocarboxylation of homostyrenyl acetates with CO_2_, providing α-aryl carboxylic acids with good selectivity and yield^16^ (Table S1,† entry 7). In this process, the catalyst Pd(OAc)_2_, need to be added to each experiment, which is expensive and difficult to recycle. Thus the development of mild allylation of CO_2_ with simple operation and low cost is highly desirable.

Metal complexes (Co, Ni, Pd)^17–19^ and silver^20–27^ respectively, have been proven to be efficient in electrosynthesis of carboxylic acids *via* carboxylation of halogenated compounds. For combining the catalytic function of both, organically doped metals^28–30^ were introduced, which used as catalysts with the advantages of high catalytic activity as well as easy separation. More importantly, it could be reused in electrosynthesis as cathode. Our research group has already reported that organically doped electrodes catalyse the reduction of CO_2_ and asymmetric electrocarboxylation of benzyl bromide with good results.^31,32^ As part of our ongoing interest in electrochemical fixation of CO_2_ with organically doped metals, we questioned whether the allylation reaction of CO_2_ to synthesis β,γ-unsaturated carboxylic acids could be achieved by organically doped metal. At the same time, we want to know whether regioselective electrocarboxylation could be realized (Scheme 1)^10^ in this reaction process. Herein, organically doped metal [Cu]@Ag was synthesized and used as cathode for electroallylation of CO_2_ with cinnamyl chloride (1a) (Scheme 1). We showed that CO_2_ allylation to β,γ-unsaturated carboxylic acids on [Cu]@Ag cathode could be carried out under mild conditions. In addition, reasonable yields and selectivities of carboxylic acids were obtained with other allylic halides (X = Cl, Br, I) and aryl halide. By the way, [Co]@Ag and Ag NPs were also prepared (see ESI†) and used in the above reaction to study the effect of cathode material.

**Scheme 1:**
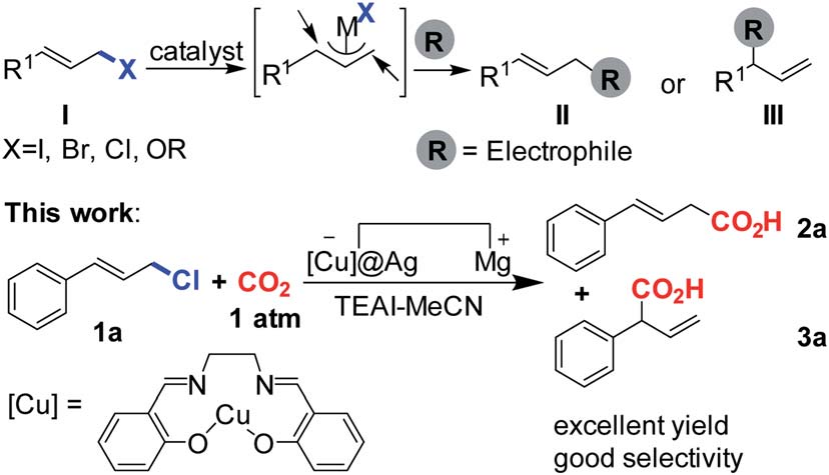
Regiodivergency in allyl electrophiles and our concept of the allylation of CO_2_.

[Cu]@Ag composite was synthesized by heterogeneous reduction with Zn as the reducing agent for silver nitrate in the present of *N*,*N*′-bis(salicylidene)ethylene diamine copper (Cu-salen) (see ESI, Scheme S1†). The morphology of the composite was investigated by SEM. The [Cu]@Ag composite consist of spheres-like nanoparticles about 70 ± 20 nm in size (Fig. 1A), which appears to have a similar size with [Co]Ag (Fig. 1B), but smaller size than pure Ag NPs (Fig. 1C). [M]@Ag composite with smaller crystal radii may be induced by the retarding of the added [M-salen] in the stage of crystal growth of silver metal. Fig. 1A–C also show that they are porous, with hierarchical structure. According to XRD, the doped [Cu]@Ag and [Co]@Ag have typical diffraction peaks of Ag NPs (Fig. 1D), almost no other characteristic diffraction peaks. Therefore, the presence of metal complex dopants in [Cu]@Ag and [Co]@Ag needs further characterization by EDAX and UV.

**Fig. 1:**
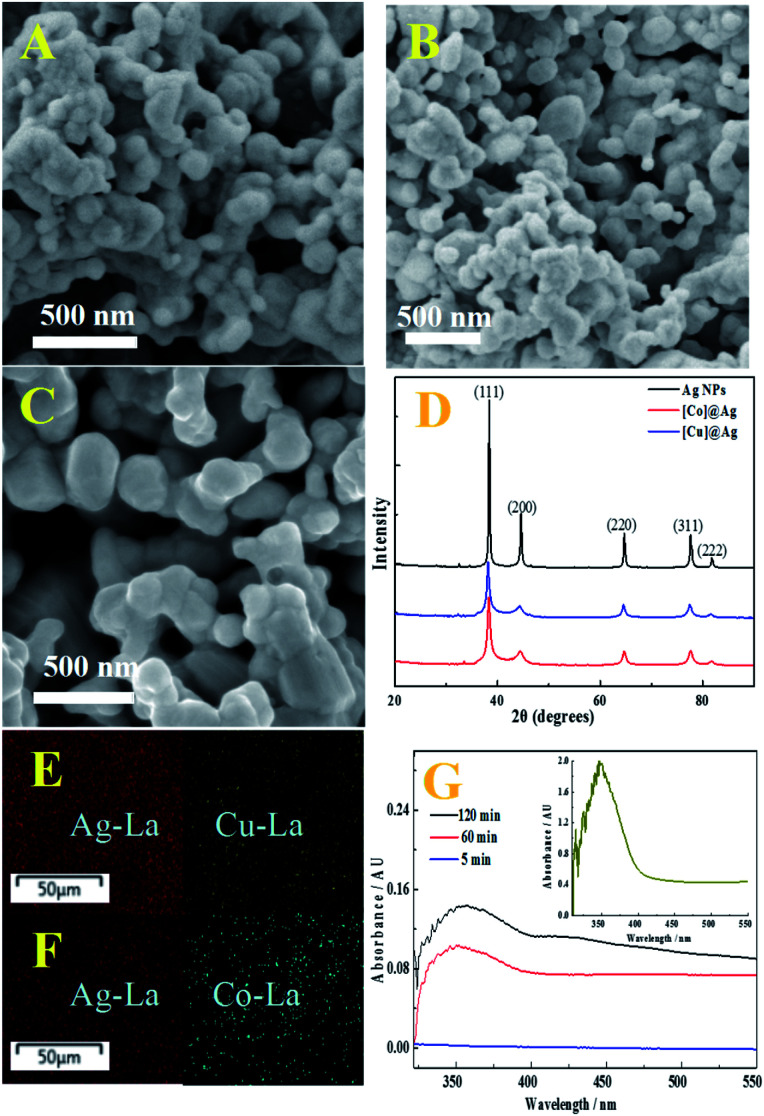
(A) SEM of [Cu]@Ag; (B) SEM of [Co]@Ag; (C) SEM of Ag NPs; (D) XRD patterns of pure Ag NPs (black), [Co]@Ag (red) and [Cu]@Ag (blue). (E) X-ray atomic mapping of [Cu]@Ag, red and yellow colours represent silver and copper atoms, respectively; (F) X-ray atomic mapping of [Co]@Ag, red and blue colours represent silver and cobalt atoms, respectively; (G) spectra of [Cu]@Ag in DMSO with different extraction time. Inset shows the spectra of Cu-salen in DMSO.

The EDAX spectrum of [Cu]@Ag and [Co]@Ag showed signals of copper (Fig. S1A†) and cobalt (Fig. S1B†), besides of silver signal. EDX mapping of [Cu]@Ag (Fig. 1E) and [Co]@Ag composites (Fig. 1F) show homogenous dispersion of the metal (copper, cobalt) complex in the composites, as indicated by different colors for copper (yellow), cobalt (blue) and silver (red).

The entrapment of Cu-salen was proved by UV indirectly. The inset of Fig. 1G displays the spectra of Cu-salen in DMSO. It is obvious that Cu-salen has a characteristic ultraviolet absorption peak at about 350 nm. The [Cu]@Ag powder was dispersed in DMSO and sonicated for 5 minutes, and it was found that there was no absorption peak at 350 nm (blue line), but as the ultrasonication time was extended, the absorption peak intensity at 350 nm was significantly increased (red and black line). It indicates that Cu-salen complex was entrapped into the composite, as well as the complex did not change during the entrapment process.

To examine the catalytic activity of [Cu]@Ag, the powder of [Cu]@Ag composite was pressed into a coin and used as cathode for electrocatalytic carboxylation of cinnamyl chloride (1a) in undivided cell under galvanostatic conditions with magnesium (Mg) as anode. 77% yield of carboxylic acids and 71% selectivity of 2a were obtained on [Cu]@Ag (Table 1, entry 1). Replacing [Cu]@Ag with DMSO-washed [Cu]@Ag gave the same selectivity but lower yield (Table 1, entry 2), which indicated that the doped Cu-salen plays an important role in the reaction. Since It is reported that Ag and Co-salen were efficient in electrocarboxylation of halides. The reactions were also carried out on pure Ag NPs and [Co]@Ag cathode. 59% yield and 70% selectivity were obtained on Ag NPs (Table 1, entry 3). In case of [Co]@Ag cathode, 63% yield and 69% selectivity were obtained (Table 1, entry 4). The results show that the selectivity of 2a remains almost unchanged, which may be due to the fact that the selected electrodes have little effect on regioselective carboxylation of cinnamyl chloride. The experimental results also show the carboxylation yields are improved by the use of [M-salen]@Ag comparing with the case of using Ag NPs (entries 1, 3 and 4). The positive effect of [M-salen]@Ag cathode may be due to the catalytic role of [M-salen] doped on the surface of Ag NPs as well as the increasing of the surface area of the cathode.

**Table 1:** Electrocarboxylation of cinnamyl chloride with CO_2_ at different cathodes^a^

Entry	Cathode	*S* of 2a (%)	Yield (2a + 3a)^b^ (%)
1	[Cu]@Ag	71	77
2^c^	[Cu]@Ag	71	67
3	Ag NPs	70	59
4	[Co]@Ag	69	63

a The reaction was carried out with Mg anode in tetraethyl ammonium iodide (TEAI)-MeCN-1a (0.05 M) saturated with CO_2_ at 25 °C with 8 mA cm^−2^ current density and 2.0 F mol^−1^ charge in undivided cell.

b Chemical yield was determined by the HPLC.

c [Cu]@Ag was washed by DMSO.

To verify our speculation, the electroreduction of cinnamyl chloride at different electrodes was studied by cyclic voltammetry (Fig. 2). According to the CV scans, an irreversible reduction peak appears at −0.9 V on [Cu]@Ag electrode under nitrogen atmosphere (curve a). It is derived from the 2e^−^ reduction of cinnamyl chloride.^21,23^ However, the 2e^−^ reduction peak of 1a at pure Ag NPs and [Co]@Ag were appeared at more negative potentials −1.3 V (curve c) and −1.1 V (curve b) respectively. [Cu]@Ag with most positive potential and highest current density indicated best catalytic activity, which agreed with our speculation.

**Fig. 2:**
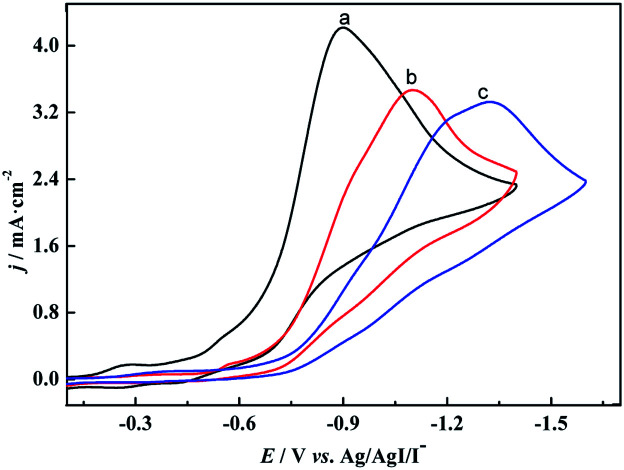
Cyclic voltammograms of 5 mM cinnamyl chloride recorded at (a) [Cu]@Ag electrode, (b) [Co]@Ag, (c) Ag NPs in 0.1 M tetraethylammonium chloride (TEACl)-MeCN solution at a sweep rate of 200 mV s^−1^ at 25 °C saturated with N_2_.

To further confirm it, the double-layer capacitance (*C*_dl_) was measured by cyclic voltammetry (CV) to evaluate the electrochemical surface area (ECSA) of the prepared electrodes. The CV curves were obtained at different scan rates (20, 60, 100, 140, and 180 mV s^−1^) with a potential window of −0.04 V to −0.2 V *versus* Ag/AgI/I^−^ (Fig. 3). The *C*_dl_ was estimated by plotting the halves of the anodic and cathodic current density at −0.12 V *versus* Ag/AgI/I^−^ against the scan rate, in which the slope was the *C*_dl_.^33,34^ As shown in Fig. 3D, the C_dl_ of [Cu]@Ag electrode is 99.1 uF cm^−2^, which is about 2.9 and 3.7 times than that of [Co]@Ag and pure Ag NPs respectively. The increase in electrochemically active surface area means the enhancement of electrocatalytic performance, which further supported our conclusion.

**Fig. 3:**
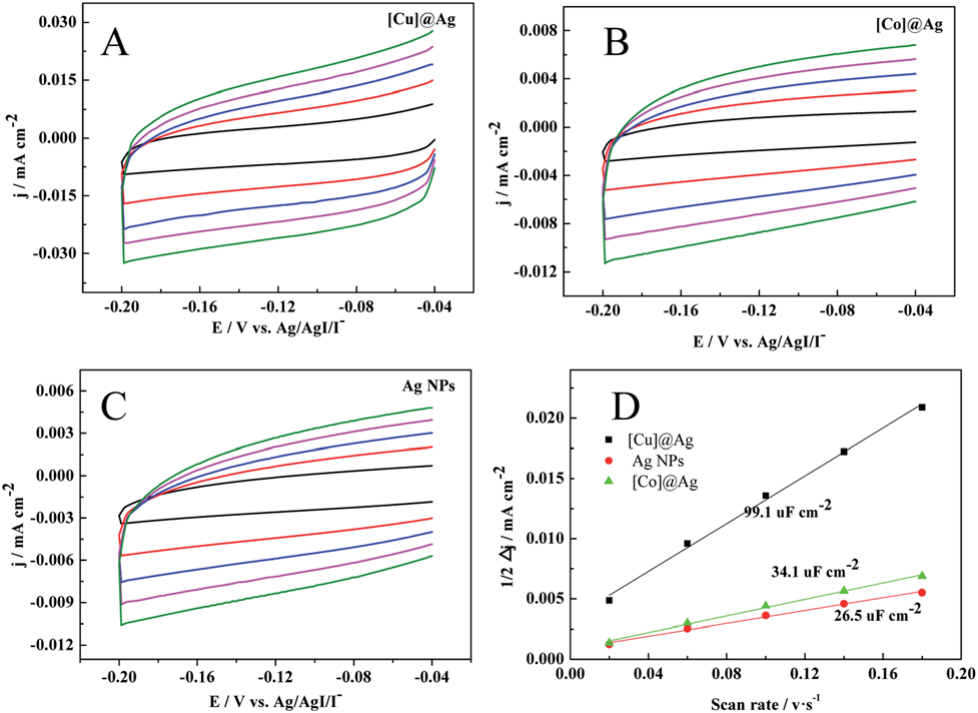
CV graphs of (A) [Cu]@Ag, (B) [Co]@Ag and (C) pure Ag NPs at scan rates of 20, 60, 100, 140, and 180 mV s^−1^ in N_2_-saturated of TEACl-MeCN-1a (5 mM) solution, and (D) plots of the current density (Δ*j* = *j*_a_ − *j*_c_) against the scan rate for [Cu]@Ag, [Co]@Ag, and Ag NPs.

To optimize the yield of carboxylic acids and the selectivity of 2a, the effects of different parameters such as supporting electrolyte, charge amount, current density and temperature on the reaction were investigated with Cu@Ag cathode (Fig. 4). To compare the performances of supporting electrolytes, a set of electrolyses was performed under the same conditions. Reasonable yields of carboxylic acids were obtained with all electrolytes including TEAI, TEABr, TEACl, TBAI, TBABr and TBACl. As shown in Fig. 4A, both the anions and cations of supporting electrolytes affect the electrocatalytic carboxylation of cinnamyl chloride. When the cations are the same, the yield of carboxylic acid decreased in order of Cl^−^ > I^−^ > Br^−^, which may be attributed to the strong adsorption of Br^−^ and I^−^ on the silver electrode.^35^ When with the same anions, TEA^+^ provided better yields than that of TBA^+^. This may be because the carboxylic acid anion produced during the electrocarboxylation process is better stabilized by TEA^+^, which is more conducive to electrosynthesis.^21^ In addition, the 2a selectivity is less affected by electrolytes, which fluctuate between 70% and 76%. As for cations, TEA^+^ provided better selectivity than that of TBA^+^. As for anions, 2a selectivity decreased in order of Cl^−^ > Br^−^ > I^−^. 82% yield of carboxylic acids and 76% selectivity of 2a were achieved with TEACl as supporting electrolytes.

**Fig. 4:**
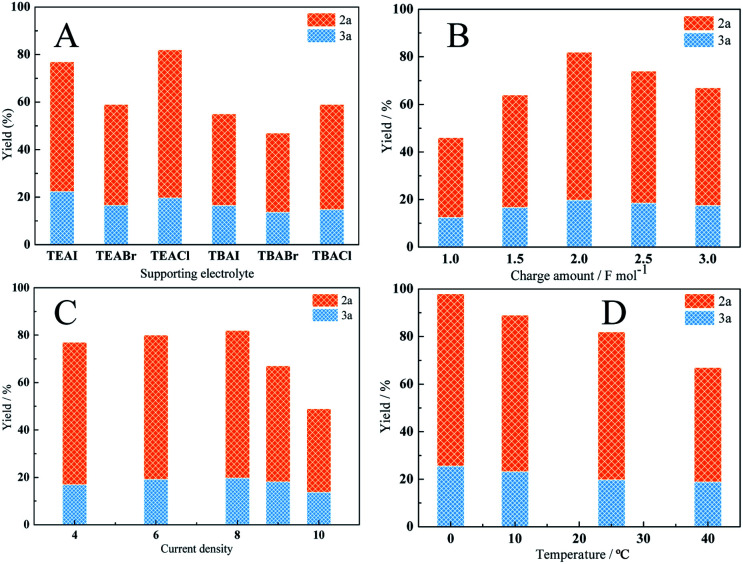
Electrocarboxylation of cinnamyl chloride on different conditions.

The charge amount also influenced the yield of carboxylic acids, but minimally affected the selectivity of product 2a (Fig. 4B). Before 2.0 F mol^−1^, the carboxylic acids yield was increased with the charge passed during electrolyses. After then, the acids yield dropped to 67% for 3.0 F mol^−1^, which may be due to the decarboxylation of the formed carboxylate.^36^

To check the influence of current density, a set of electrolyses was performed in the range of 4 mA cm^−2^ to 10 mA cm^−2^. As shown in Fig. 4C, both high and low current densities led to lower carboxylic acid yields. It may be due to the corresponding potential at low current density is relatively positive, which may be unfavorable for the electroreduction of the cinnamyl chloride. While the corresponding potential at high current density is negative, and the reduction of cinnamyl chloride may be accompanied by the reduction of CO_2._ The best yield (82%) was obtained at 8 mA cm^−2^. It's worth noting that the selectivity of 2a (78%) is slightly better at low current density (4 mA cm^−2^). This may be due to the slow reaction rate at low current density, which promotes the reaction of allyl anions (PhCH=CHCH_2_^−^) generated by electroreduction with CO_2_.

Temperature affects the solubility of CO_2_ in MeCN as well as the kinetics of the reaction. In general, low temperature is conducive to the dissolution of CO_2_, and high temperature is beneficial to the kinetic reaction. Therefore the reactions were carried out in the range of 0–40 °C to examine the effects of the reaction temperature. As shown in Fig. 4D, the total yield of carboxylic acid increases as the reaction temperature decreases. The highest total yield 98% and moderate selectivity 76% were achieved at 0 °C. In order to achieve the better selectivity, the same reactions was carried out under −10 °C with different solvents such as MeCN, DMF and DMA (Table 2). Unfortunately, the selectivity of 2a increased slightly. According to the literature,^10^ the ligands of catalysts have a great influence on regioselectivity, which will be studied in our future work to improve the regioselectivity of the reaction.

**Table 2:** Electrocarboxylation of cinnamyl chloride on [Cu]@Ag in different solvents under low temperature^a^

Solvent	*T* (°C)	3a : 2a^b^	Yield (2a + 3a)^c^ (%)
MeCN	−10	21/79	98
DMF^d^	−10	18/82	75
DMA^e^	−10	22/78	27

a The reaction was carried out in undivided cell with Mg anode and 1 mmol of TEACl in 10 mL of solvent saturated with CO_2_.

b The ratio of regiomers was determined by the HPLC.

c Chemical yield.

d DMF = *N*,*N*-dimethylformamide.

e DMA = *N*,*N*-dimethylacetamide.

To examine the effectiveness of the catalytic electrode [Cu]@Ag, the reaction proceeds with cinnamyl bromide (1b), allyl chloride (1c), allyl bromide (1d), allyl iodide (1e), 3-chloro-1-butene (1f) and 3-chloro-2-methylpropene (1g) under the optimal conditions (Table 3). When 1b was used as substrate (Table 3, entry 2), the electrolysis results are similar to 1a (entry 1). Under the same conditions, allyl halides 1c–1e afforded 2c with 56%, 54%, and 37% yields respectively (entries 3–5). Compared with the cinnamyl halides, carboxylation yield of allyl halides was much lower. In order to explore the reasons, the reduction of allyl halides (taking allyl chloride 1c as an example) was studied by CV, which was compared with that of cinnamyl chloride. As shown in Fig. 5, no redox peaks appeared in the scan region before the substrate was added under N_2_ atmosphere (curve a). After addition of 5 mM 1c, an irreversible reduction peak at −1.47 V, more negative than that of cinnamyl chloride (curve c), ascribed to 2e^−^ reduction of 1c was observed (curve b). This indicates that allyl chloride is less susceptible to electroreduction under optimal conditions of cinnamyl chloride. Namely, the conversion of allyl halides is lower than that of cinnamyl halides, which is disadvantageous for electrocarboxylation. Thus lower yields of carboxylic acids were obtained using allyl halides as the starting materials.

**Table 3:** Electrocatalytic carboxylation of other allylic halides under optimized conditions^a^

Entry	Substrate		Product		Carboxylation yield^b^(%)	S of 2 (%)
1	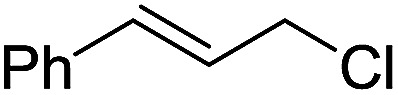	1a	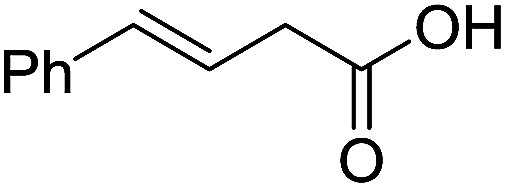	2a	98	76
2	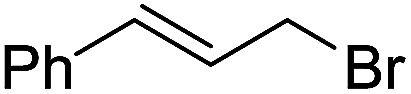	1b			99	75
3	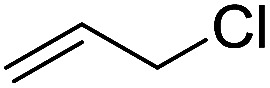	1c	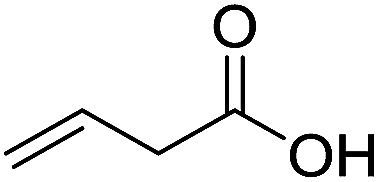	2c	56	100
4	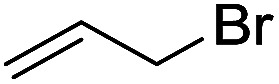	1d			54	100
5	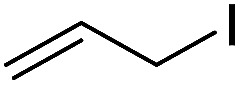	1e			37	100
6	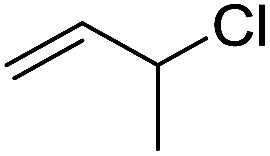	1f	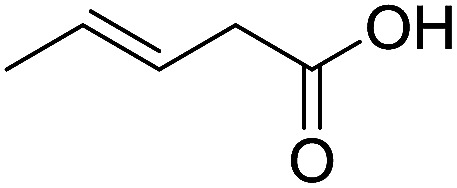	2f	48	76
7	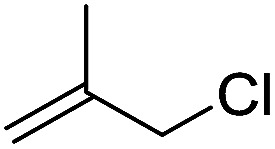	1g	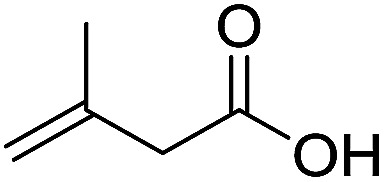	2g	51	100

a The reaction was carried out in undivided cell with [Cu]@Ag cathode, Mg anode and 1 mmol of TEACl 0.05 M substrate in 10 mL of MeCN saturated with CO_2_ at 0 °C with 8 mA cm^−2^ current density and 2.0 F mol^−1^ charge.

b Chemical yield was determined by the HPLC.

**Fig. 5:**
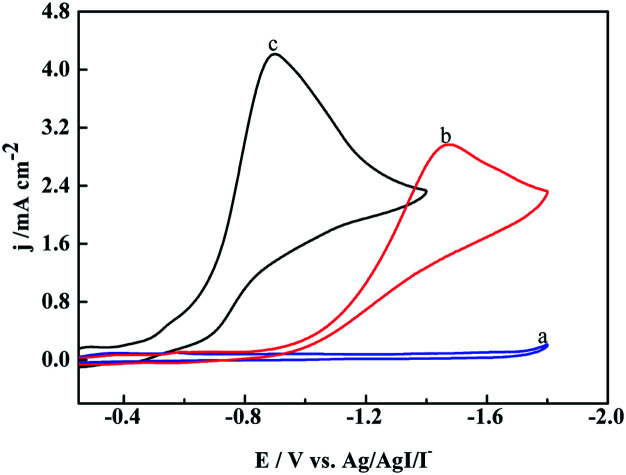
Cyclic voltammograms of (a) TEACl-MeCN; (b) 5 mM allyl chloride; (c) 5 mM cinnamyl chloride recorded at [Cu]@Ag in 0.1 M TEACl–MeCN solution at a sweep rate of 200 mV s^−1^ at 25 °C saturated with N_2_.

Halogen atoms (X) in the substrates 1c–1e also affect the yield of carboxylic acid. It is well known that iodides are more susceptible to nucleophilic substitution than chlorides.^25^ So, iodides may generate dimerization products much easier under the same electrolytic conditions, resulting in lower carboxylation yield (entry 5).

To study the effect of the substituent position on the electrocarboxylation, two isomers 3-chloro-1-butene (1f) and 3-chloro-2-methylpropene (1g) were used as substrate, and the carboxylation yields were similar (entries 6 and 7). In case of 1g, only methyl-3-butenoic acid (2g) was obtained. But, in case of 1f, the mixture of 3-pentenoic acid (2f) and 2-methyl-3-butenoic acid (3f) were obtained.

In addition, the electrocatalytic carboxylation of aryl chloride on [Cu]@Ag electrode was also attempted. The corresponding carboxylic acid could be obtained with excellent yield when using 4-vinylbenzyl chloride (1h) as a substrate (Scheme 2).

**Scheme 2:**
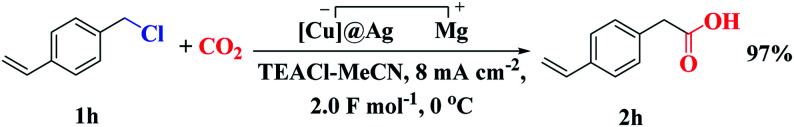
Electrocatalytic carboxylation of aryl chloride.

The regioselectivity in this work can be rationalized by considering the configuration of the allyl anion generated by reduction of allylic halides. The allyl anion has two electrons in the nonbonding orbital, giving half a negative charge to each of C1 and C3, which is consistent with the resonance forms and our optimized structure (Scheme 3). The addition of allyl anion of two resonance forms to CO_2_ gives the same carboxylate anions. This can explain that when the substrates are 1c–1e (Table 3, entries 3–5), the selectivity of carboxylic acid is 100%. When methyl substitutes for a hydrogen atom on C2, its regioselectivity remains 100%, because it does not affect configuration of the allyl anion and its charge distribution (Table 3, entry 7). In case of cinnamyl anion, the hydrogen atom on C3 is substituted by a phenyl group, the two resonance forms have different reactivity with CO_2_, which mainly depends on the stability of the carbon anion. Secondary allylic carbanion is much more destabilized than the corresponding primary allylic carbanion,^37^ thus the regioselectivity of 2a is much higher than 3a (Table 3, entries 1 and 2). A methyl substitution of the hydrogen atom on C1 is similar to cinnamyl anion. More stabilized allylic carbanion in the presence of CO_2_ gives more corresponding carboxylic acid (Table 3, entry 6). In addition, the energy of the products (2a, 3a, 2f and 3f) in gas phase was calculated by density functional theory (DFT) B3LYP/6-31+G(d,p) method (Table S2†). Comparing the energy of the two products 2a/f and 3a/f corresponding to the substrates 1a/f respectively, the energy of 2 is lower than 3. That is, 2 is more stable. The results further illustrate the rationality of the regioselectivity.

**Scheme 3:**
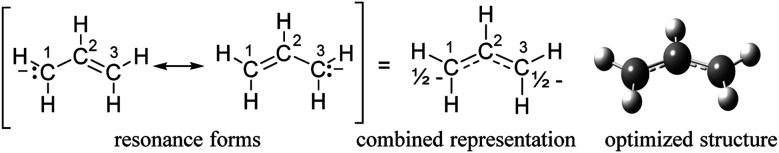
The resonance forms of allyl anion.

Based on the previous work on electrocarboxylation of organic halides catalyzed by cobalt salen complexes^17,38^ and our observation, a possible mechanism of CO_2_ electroallylation with cinnamyl chloride on [Cu]@Ag electrodes was proposed (Scheme 4). Firstly, nucleophilic Cu(i)salen^−^ generated from the one-electron reduction of Cu(ii)salen encapsulated in the [Cu]@Ag cathode, which reacts with cinnamyl chloride (1a) to form Cu(iii)salen–CH_2_CH=CHPh (1). Then, complex 1 is directly reduced at the [Cu]Ag cathode to regenerate Cu(ii)salen and form cinnamyl anion (2) by one-electron transfer. Subsequently, 2 isomerization formed terminal olefin anion 3. In the presence of CO_2_, corresponding carboxylates were formed, and the carboxylic acids 2a and 3a could be obtained by the treatment of hydrochloric acid.

**Scheme 4:**
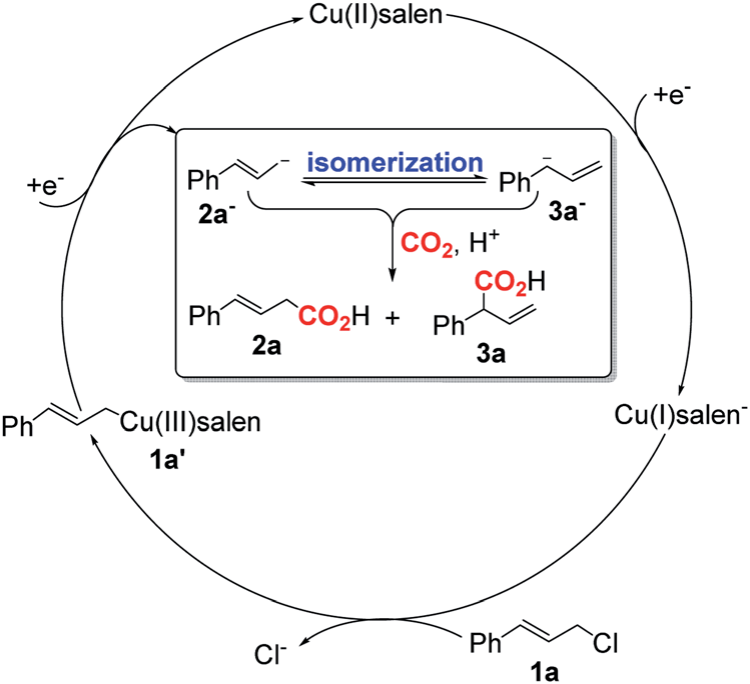
Proposed reaction mechanism of CO_2_ electroallylation at [Cu]@Ag cathode.

[Cu]@Ag was successfully synthesized by the entrapment of Cu-salen complex within silver. It was used as cathode to investigate CO_2_ electroallylation with cinnamyl chloride, corresponding carboxylic acid was achieved with excellent yield and moderate selectivity. This work expanded the application of doped metals as cathode in CO_2_ fixation, as well as provided a green, mild and efficient way for the preparation of β,γ-unsaturated carboxylic acids with important applications.

## Conflicts of interest

There are no conflicts of interest to declare.

## Supplementary Material

RA-009-C9RA05253D-s001
