# Supplementary material for: Silver encapsulated copper salen complex: efficient catalyst for electrocarboxylation of cinnamyl chloride with CO2
Source: RSC Adv. 2019 Oct 15;9(56):32628–33. doi: 10.1039/c9ra05253d (PMC9073196; doi:10.1039/c9ra05253d)
Supplement: RA-009-C9RA05253D-s001 [file RA-009-C9RA05253D-s001.pdf]

Electronic Supplementary Material (ESI) for RSC Advances.
This journal is © The Royal Society of Chemistry 2019

Supporting Information

Silver encapsulated copper salen complexes: Highly efficient catalyst for electrocarboxylation of cinnamyl chloride with CO_2

La-Xia Wu[a], Ying-Guo Zhao[a], Ye-Bin Guan[a], Hui Wang[b], Yang-Chun Lan[c], Huan Wang[*b], Jia-Xing Lu[**b]

[a]AnHui Province Key Laboratory of Functional Coordination Compounds, School of Chemistry and Chemical Engineering, Anqing Normal University, Anqing 246011, China.

[b]School of Chemistry and Molecular Engineering, East China Normal University, Shanghai 200062, China.

[c]Department of Electrical and Electronic Engineering Southern University of Science and Technology, ShenZhen 518055, China.

[*]Corresponding author. Tel: +86-21-52134935; E-mail: hwang@chem.ecnu.edu.cn

[**]Corresponding author. Tel: +86-21-62233491; E-mail: jxlu@chem.ecnu.edu.cn

Table of Contents

1. Materials and Instrument..........2

2. General procedure..........2

2.1 Preparation of cathodes..........2

2.2 Electrochemical study..........3

3. Summary and comparison of different ways to synthesize β,γ-unsaturated carboxylic acids..........4

4. EDX Spectrum of [Cu]@Ag and [Co]@Ag..........4

5. Energy of product in gas phase..........5

6. NMR characterization..........5

References..........10

1. Materials and Instrument

Galvanostatic electrosynthesis was performed using a digital direct current-regulated power supply (HY3005MT, HYelec® China). Voltammetric measurements were conducted using electrochemical station (CHI650C, Chenhua) in a conventional three-electrode cell. The product yield was determined by high-performance liquid chromatography (HPLC) instrument (DIONEX Ultimate 3000 pump) (Thermo Scientific, Germering, Germany) equipped with a UV (RS Variable Wavelength) (Thermo Scientific, Germering, Germany) detector. Microstructure and morphology of [M]@Ag were analyzed using Hitachi S4800 field-emission scanning electron microscope (FE-SEM) (Hitachi, Tokyo, Japan) equipped with an energy dispersive X-Ray fluoresence spectrometer (Ametek, Oxford, UK). X-ray diffraction (XRD) (Rigaku Corporation, Tokyo, Japan) patterns were recorded by a Ultima IV X-ray powder diffractometer using Cu K_{α} radiation (k = 1.5406 Å). ^{1}HNMR spectra were recorded on an AVANCE 500 (500 MHz, Bruker, Germany) spectrometer in $CDCl_3$ with Me_4Si as an internal standard. The calculation of energy was performed in Gaussian 09 D.01 using B3LYP method with 6-31+G(d,p) set. All substrates, vinylacetic acid (**2d**) and Co-salen were used as received (Energy chemical, China). MeCN were kept over 4 Å molecular sieves.

2. General procedure

2.1 Preparation of cathodes

The synthesis process of [M]@Ag cathode was shown in Scheme S1.

Synthesis of [Cu]@Ag: In typical procedure, 16 mg Cu-salen prepared as described in literature[1] and 0.5 g sodium dodecyl sulfate (SDS) were added into a solution of 0.85 g $AgNO_3$ and 50 mL distilled water. After stirring for 30 min, 0.17 g zinc powder was slowly added into the reaction system and then the solution was stirred for 8 hours at room temperature. The product was collected by centrifuging, washed with 5 mL of 1 M HCl for 2 times to remove remaining zinc powder, then washed with distilled water and ethanol for many times and dried under vacuum overnight. Finally, the powder was pressed into a coin (d = 2 cm) and used as cathode.

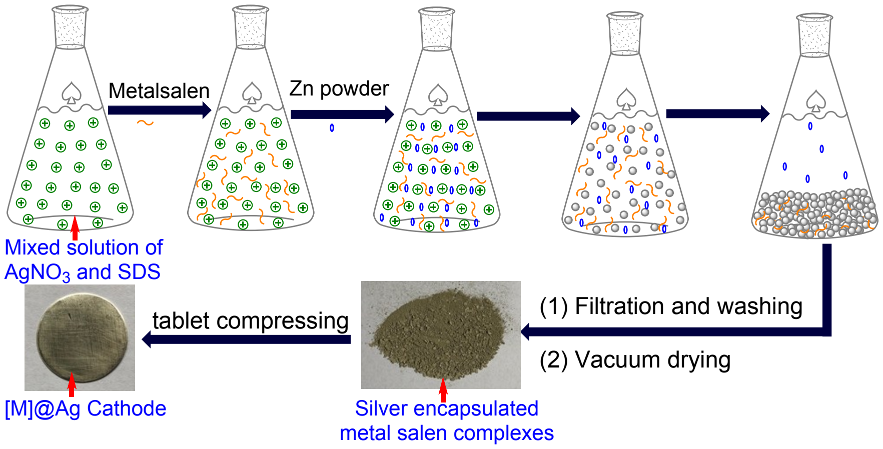

Scheme S1. Synthesis Process of [M]@Ag cathode

[Co]@Ag and Ag NPs were prepared in the same way. In case of [Co]@Ag, 16 mg of Co-salen was added into the reaction system instead of Cu-salen. In case of Ag NPs, no metal salen complex was added.

2.2 Electrochemical study

Electrochemical measurements were performed in a conventional three-electrode cell at a electrochemical workstation (CHI 650C). Typically, 10 mg of synthetic powder was suspended in the mixture solution of 40 uL Nafion solution (5 wt%), 750 uL deionized water and 250 uL ethanol, and a homogeneous ink was formed by ultrasound for 1 h. Then, 6 uL of the ink was loaded onto a glassy carbon electrode (GC) with 1 mm radius. The electrochemical behavior of **1a** was scanned at different cathodes ([Cu]@Ag, [Co]@Ag, Ag NPs) in tetraethylammonium chloride-acetonitrile (TEACl-MeCN) solution with a sweep rate of 0.2 V s^{-1} in a nitrogen atmosphere. The counter and the reference electrodes were the platinum sheet (1 cm × 2 cm) the Ag/AgI/0.1 mol L^{-1} TBAI respectively.

Typical electrocatalytic carboxylation was conducted in a mixture of 50 mM cinnamyl chloride (**1a**), 0.1 M TEACl and 10 mL MeCN saturated with CO_2 (1 atm) in an undivided cell equipped with [Cu]@Ag cathode and Mg rod anode in One-compartment electrolytic cell. During the electrolysis process, CO_2 bubbles are always bubbling into the reaction system. The electrolysis was stopped after the consumption of 2.0 F/mol of **1a**. At the end of the electrolysis, MeCN was removed under reduced pressure, the rest was hydrolyzed with hydrochloric acid (0.2 M, 15 mL) and extracted with Et_2O (15 mL) for 4 times. Combined ethereal layers and dried with $MgSO_4$ for 4 h, after then remove the solvent under reduced pressure, products **2a** and **3a** were purified using flash chromatography (Petroleum ether-ethyl acetate 16:1) and characterized by ^{1}HNMR and ^{13}CNMR. The yields were determined by high-performance liquid chromatography (HPLC).

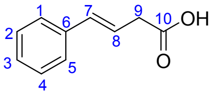

(*E*)-4-*Phenyl-3-butenoic acid 2a*

^{1}H NMR (500 MHz, $CDCl_3$) δ 7.43-7.38 (m, 2H), 7.34 (t, J = 7.6 Hz, 2H), 7.28-7.24 (m, 1H), 6.55 (d, J = 15.9 Hz, 1H), 6.31 (dt, J = 15.9, 7.1 Hz, 1H), 3.33 (dd, J = 7.1, 1.4 Hz, 2H). ^{13}C NMR (101 MHz, $CDCl_3$) δ 178.24 (C-10), 136.66 (C-6), 134.05 (C-7), 128.60 (C-2, C-4), 127.74 (C-3), 126.37 (C-1, C-5), 120.80 (C-8), 38.08 (C-9) ppm.

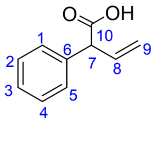

2-*phenyl-3-butenoic acid 3a*

^{1}H NMR (500 MHz, $CDCl_3$) δ 7.44 -7.29 (m, 5H), 6.24 (ddd, J = 17.7, 10.2, 8.0 Hz, 1H), 5.40-5.05 (m, 2H), 4.36 (d, J = 8.0 Hz, 1H). ^{13}C NMR (101 MHz, $CDCl_3$) δ 178.71 (C-10), 137.40 (C-6), 135.00 (C-8), 128.86 (C-2, C-4), 128.15 (C-1, C-5), 127.67 (C-3), 118.15 (C-9), 55.58 (C-7) ppm.

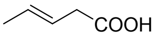

(*E*)-*3-Pentenoic acid 2f*[2,3]

^{1}H NMR (400 MHz, $CDCl_3$) δ 9.55 (s, 1H), 5.82-5.40 (m, 2H), 3.12 (dd, J =6.9, 6.6 Hz, 2H), 1.79-1.56 (m, 3H) ppm.

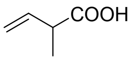

2-*Methyl-3-butenoic acid 3f*[4]

^{1}H NMR (400 MHz, $CDCl_3$) δ 10.69 (s, 1H), 5.94 (ddd, J = 17.4, 10.3, 7.4 Hz, 1H), 5.27-5.09 (m, 2H), 3.19 (pt, J = 7.1, 1.2 Hz, 1H), 1.31 (d, J = 7.0 Hz, 3H) ppm.

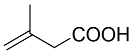

3-*Methyl-3-butenoic acid 2g*[5]

^{1}H NMR (500 MHz, $CDCl_3$) δ 4.98 (p, *J* = 1.6 Hz, 1H), 4.91 (t, *J* = 1.5 Hz, 1H), 3.11 (d, *J* = 1.2 Hz, 2H), 1.86 (s, *J* = 1.1 Hz, 3H) ppm.

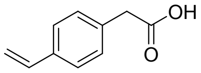

4-Vinylbenzeneacetic acid 2h[6]

^{1}H NMR (500 MHz, $CDCl_3$) δ 7.43-7.25 (m, 3H), 7.25-7.10 (m, 1H), 6.72 (dd, J = 17.6, 10.9 Hz, 1H), 5.77 (ddd, J = 17.6, 8.9, 0.9 Hz, 1H), 5.27 (ddd, J = 10.9, 9.0, 0.9 Hz, 1H), 3.67 (d, J = 3.2 Hz, 2H) ppm.

3. Summary and comparison of different ways to synthesize β,γ-unsaturated carboxylic acids

Table S1 Summary and comparison of different methods for the preparation of β,γ-unsaturated carboxylic acids

Entry	Model substrate	Catalyst	Reductant	Yield /% (L:B)[a]	Ref
1	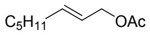	$NiBr_2$·glyme	Mn＆Zn (2.4 equiv.)	77 (99:1)	7
2	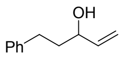	$Ni(acac)_2$	Mn (2.0 equiv.)	88 (L)	8
3	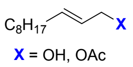	$PdCl_2$	Et_2Zn (3.0 equiv.)	94 (B)	9
4	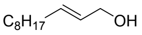	$NiBr_2$·glyme	Zn (4 equiv.)	70 (99:1)	10
5	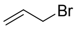	-	In (0.67equiv.)	72 (L)	11
6	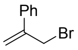	-	Zn (1.05 equiv.)	87 (B)	12
7	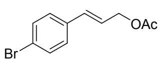	$Pd(OAc)_2$	Electric current	85 (1:20)	13
8	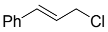	[Cu]@Ag electrode	Electric current	98 (3:1)	This work

[a] L= Linear structure, B = Branch structure.

4. EDX Spectrum of [Cu]@Ag and [Co]@Ag

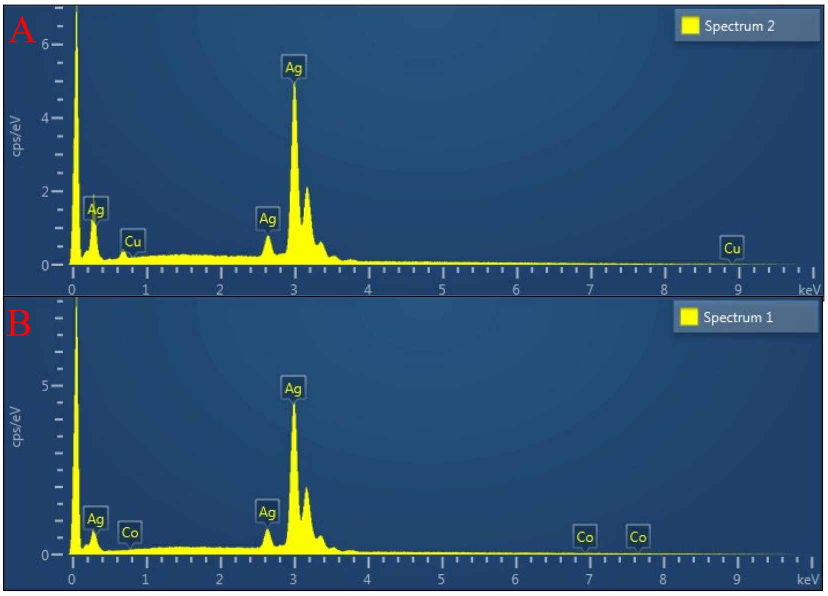

Figure S1. EDX Spectrum of [Cu]@Ag (A); [Co]@Ag composite (B)

5. Energy of product in gas phase

Table S2 Energy of product in gas phase[a]

Entry	Product		Energy/Hartree[b]
1	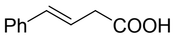	**2a**	-537.573
2	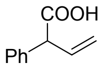	**3a**	-537.562
3	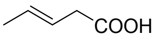	**2f**	-345.825
4	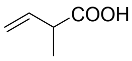	**3f**	-345.819

[a]: Calculations were performed in Gaussian 09 D.01 using B3LYP method with 6-31+G(d,p) set.

[b]: 1 Hartree = 627.51 Kcal mol^{-1}.

6. NMR characterization

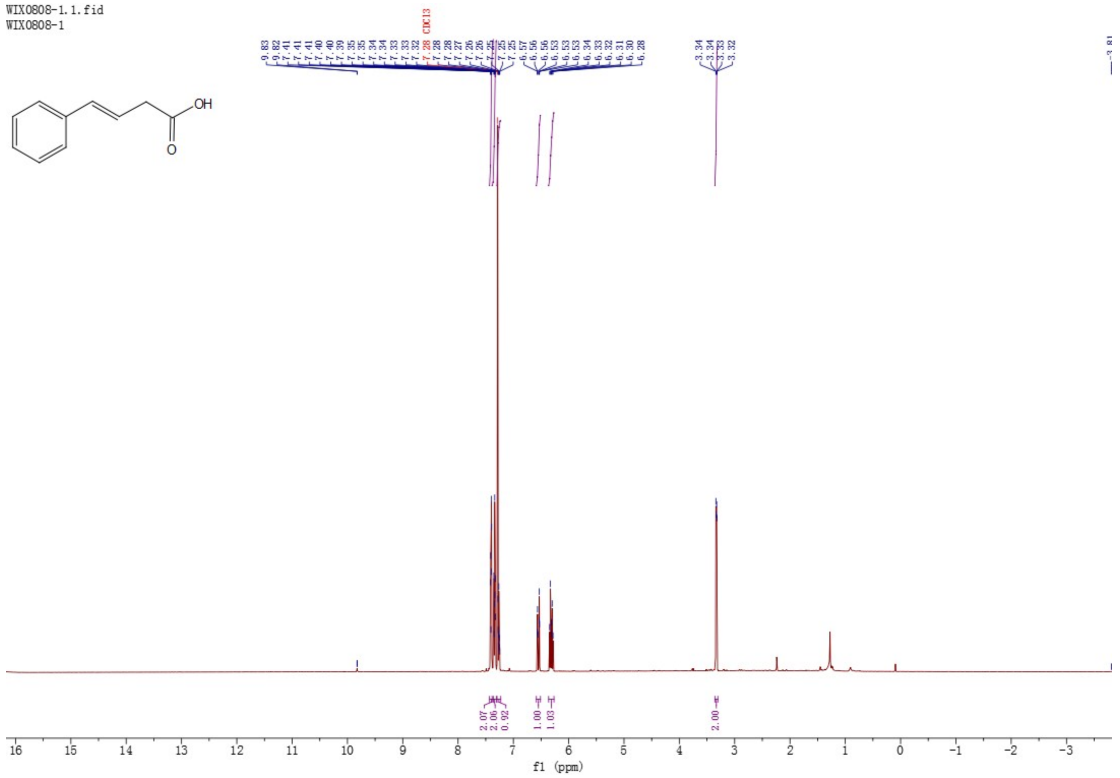

^{1}HNMR (500 MHz, $CDCl_3$) of (*E*)-phenyl-3-butenoic acid **2a**

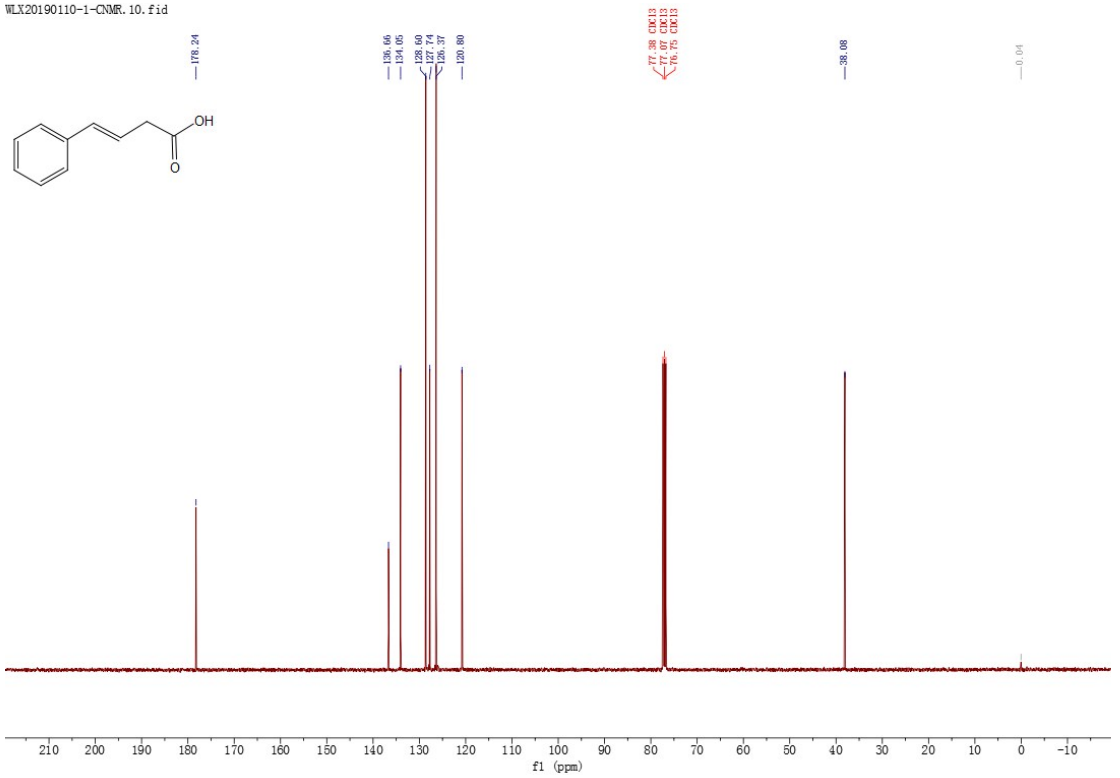

^{13}CNMR (101 MHz, $CDCl_3$) of (*E*)-phenyl-3-butenoic acid **2a**

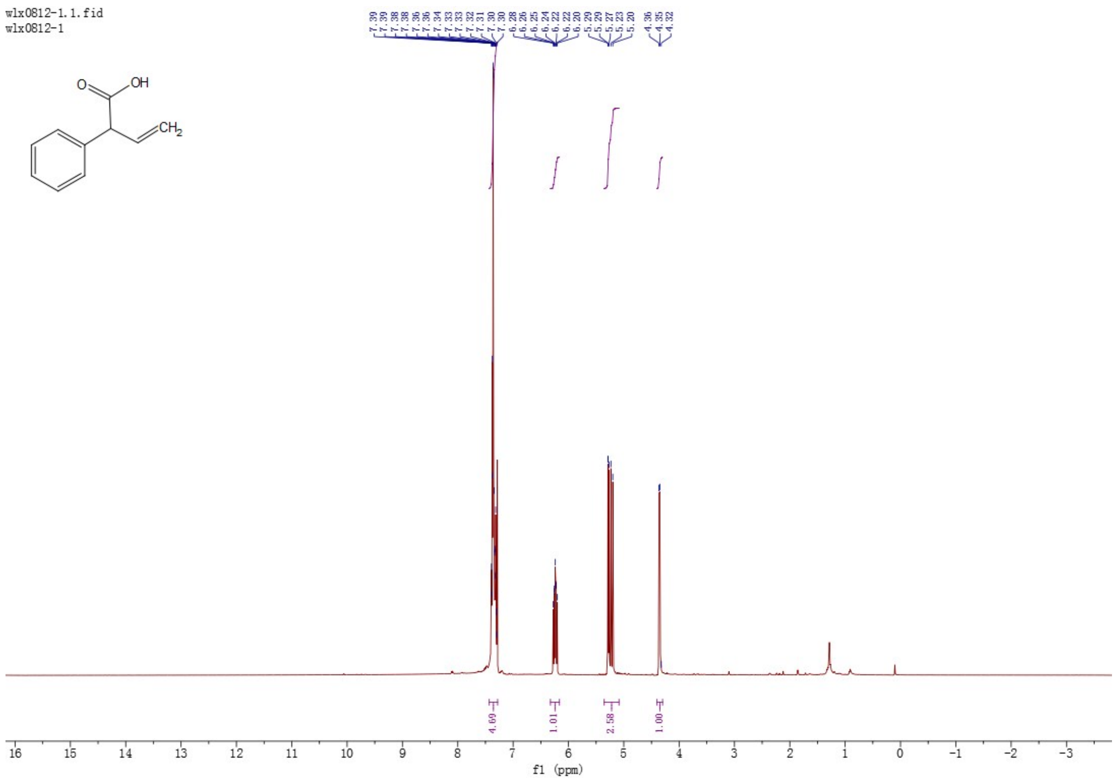

^{1}HNMR (500 MHz, $CDCl_3$) of 2-phenyl-3-butenoic acid **3a**

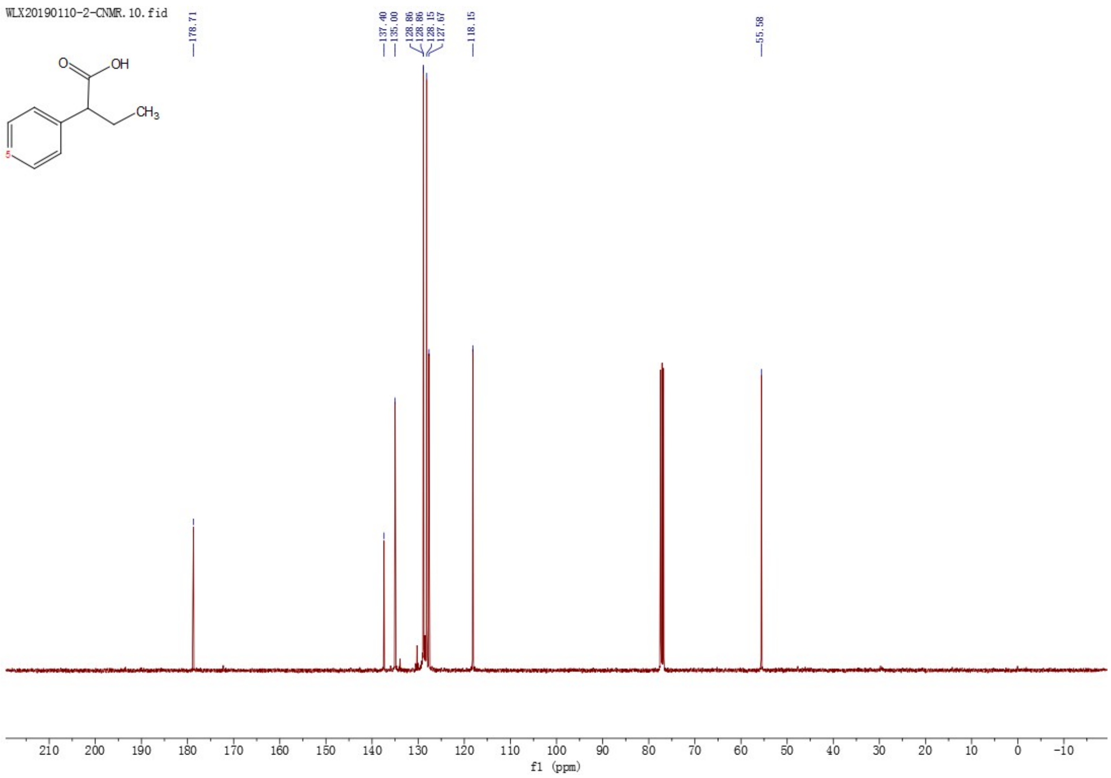

^{13}CNMR (101 MHz, $CDCl_3$) of 2-phenyl-3-butenoic acid **3a**

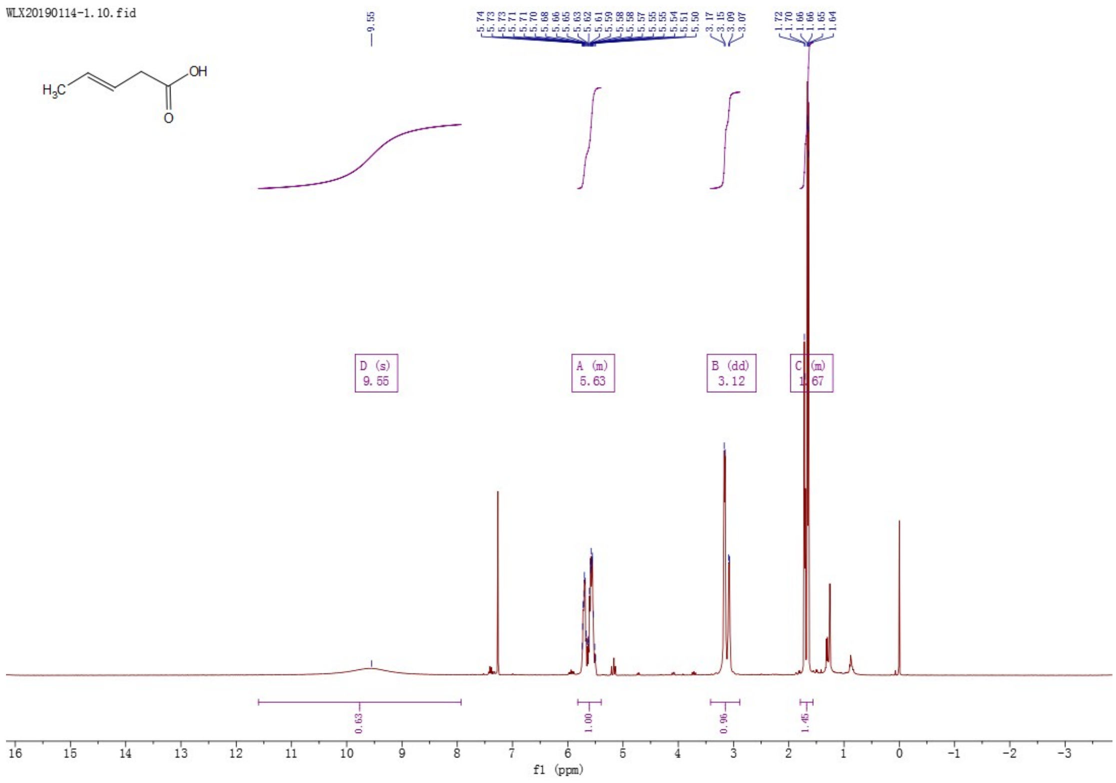

^{1}HNMR (400 MHz, $CDCl_3$) of (*E*)-3-pentenoic acid **2f**

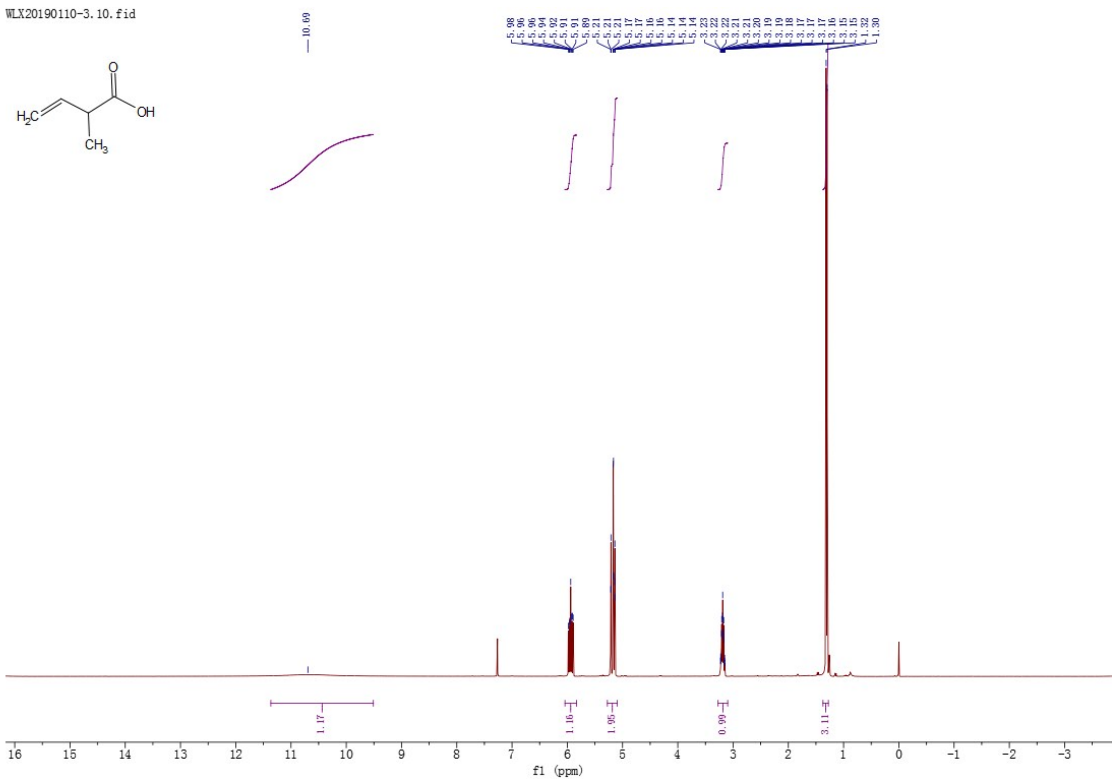

^{1}HNMR (400 MHz, $CDCl_3$) of 2-Methyl-3-butenoic acid **3f**

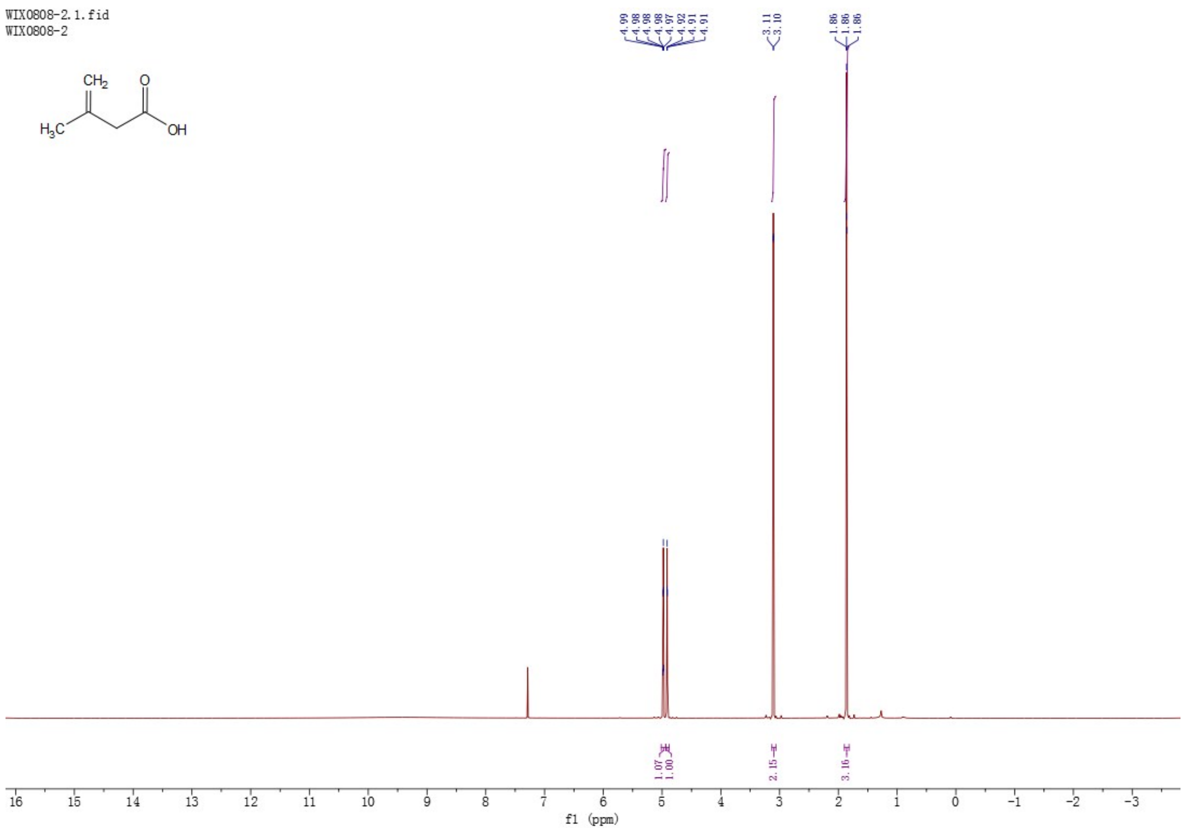

^{1}HNMR (500 MHz, $CDCl_3$)3-Methyl-3-butenoic acid **2g**

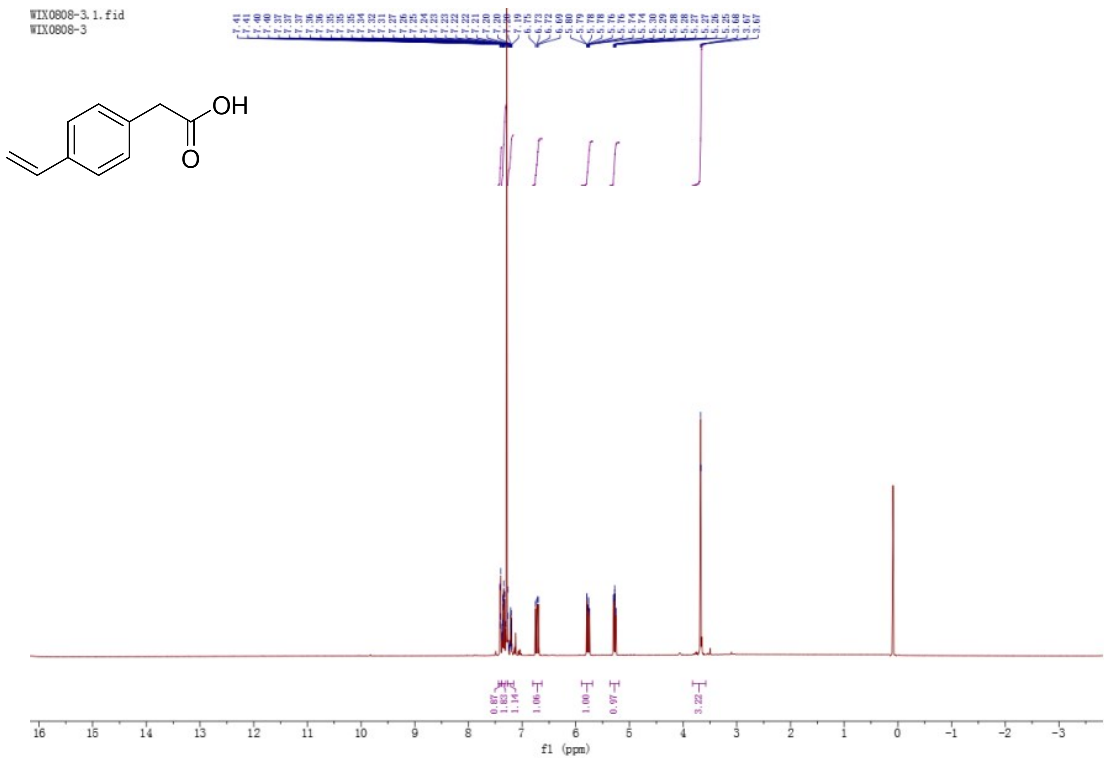

^{1}HNMR (500 MHz, $CDCl_3$) 4-Vinylbenzeneacetic acid **2h**

Reference

1. X. B. Lu, X. J. Feng and R. He, *Appl Catal a-Gen*, 2002, **234**, 25-33.

2. J. Thibonnet, M. Abarbri, J.-L. Parrain and A. Duchêne, *Tetrahedron*, 2003, **59**, 4433-4441.

3. S. J. Zhang and W. X. Hu, *Synthetic Communications*, 2010, **40**, 3093-3100.

4. H. A. Duong, P. B. Huleatt, Q. W. Tan and E. L. Shuying, *Org Lett*, 2013, **15**, 4034-4037.

5. D. F. Niu, L. P. Xiao, A. J. Zhang, G. R. Zhang, Q. Y. Tan, J. X. Lu, *Tetrahedron*, 2008, **64**, 10517–10520.

6. S. Zhang, W. Q. Chen, A. Yu and L. N. He, *Chemcatchem*, 2015, **7**, 3972-3977.

7. T. Moragas, J. Cornella, R. Martin, *J. Am. Chem. Soc.* 2014, **136**, 17702-17705.

8. Y.G. Chen, B. Shuai, C. Ma, X.J. Zhang, P. Fang, T.S. Mei, Org. Lett. 2017, **19**, 2969-2972.

9. T. Mita, Y. Higuchi, Y. Sato, *Chem. Eur. J.* 2015, **21**, 16391-16394.

10. M. van Gemmeren, M. Borjesson, A. Tortajada, S.Z. Sun, K. Okura, R. Martin, *Angew Chem Int Ed Engl* 2017, **56**, 6558-6562.

11. B. Miao, S. Ma, *Chem Commun,* 2014, **50**, 3285-3287.

12. B. Miao, S. Ma, *Org. Chem. Front.* 2015, 2, 65-68.

13. K. J. Jiao, Z. M. Li, X. T. Xu, L. P. Zhang, Y. Q. Li, K. Zhang, T. S. Mei, *Org. Chem. Front.* **2018**, 5, 2244-2248.
